# The successful experience of gymnastics world champion coach: an interview analysis

**DOI:** 10.3389/fpsyg.2024.1405589

**Published:** 2024-07-18

**Authors:** Xiuxia Liu, Xinghe Weng, Huahui Qin, Shuai Ma, Guoqing Wang

**Affiliations:** ^1^Department of Physical Education, Xiamen University, Xiamen, China; ^2^School of Psychology, Beijing Sports University, Beijing, China; ^3^Chinese Academy of Fiscal Sciences, Beijing, China; ^4^Gymnastics Center of the General Administration of Sport of China, Beijing, China

**Keywords:** gymnastic, world champion, Olympic champion, coaching success, coaching methodologies

## Abstract

**Objectives:**

The role of a coach in enhancing athletes’ performance and achieving success is well-documented across numerous studies. However, the strategies employed by Chinese coaches in developing world champion gymnasts remain under explored.

**Methods:**

This research involved a single case study focusing on a coach from the Chinese National Men’s Gymnastics Team, credited with nurturing eight world champion gymnasts.

**Results:**

The inductive content analysis leads to that 6 subthemes, “international perspective and collaborative ability,” “ability to control and regulate training loads,” “identifying athletes’ needs and transforming them into motivation,” “goal setting aligned with athletes’ abilities,” “adopting authoritative democratic coaching style,” and “establishing hierarchical-style friendship”, and 3 themes, “training management and planning,” “motivation and goal setting,” and “interpersonal communication” are manifested. An overarching theme “the successful experience of gymnastics world champion coach,” is derived from the analysis.

**Conclusion:**

This research bridges the gap between theoretical knowledge and practical application, offering valuable insights into the successful experiences of gymnastics world champion coaches. The findings have the potential to influence coaching methodologies globally, fostering the development of resilient, motivated, and high-performing athletes. Future research should focus on sport-specific studies, longitudinal analyses, and cross-cultural comparisons to further advance the field of sports coaching and validate the effectiveness of these innovative coaching strategies.

## 1 Introduction

The success of Olympic champion coaches is a testament to their multidimensional, integrated, and individualized approach, with psychological factors playing a paramount role. Interpersonal support, particularly from coaches, is crucial (Gould et al., 2002; Nash and Sproule, 2009; Breeman et al., 2014; Burns et al., 2018). Coaching facilitates the development of cognitive, affective, and learning capabilities, aimed at fulfilling personal and organizational objectives (Berg, 2006). It is essential for coaches to understand the team’s requirements and the motivational preferences of its members, applying suitable coaching methods within a supportive, transparent, and collaborative framework (Weinberg and Gould, 2015).

Effective leadership in coaching, adaptable to varying individual and situational contexts, is vital for motivating athletes toward success. This includes cultivating a productive coach-athlete relationship, enhancing motivation, and facilitating the achievement of team objectives (Kim and Cruz, 2016). Researchers have investigated the influence of coaching on superior athletic performance from two primary theoretical angles: coaching style and the coach-athlete relationship.

At the core, a successful coach assists individuals in achieving their personal and professional goals. By providing insights, tools, and techniques tailored to each person’s unique challenges, success coaches bring clarity and direction. They support athletes at every step, whether it be in business success, relationship success, financial success, or more (Gilbert and Trudel, 2004).

When coaches watch our athletes compete as the best in the world at the Olympics, we are proud of what we as a country cultivate. However, Olympic coaches, though often less visible, play an indispensable role. While defining what it means to be an effective coach is challenging and controversial, most agree that reaching the Olympic coaching level is the pinnacle of one’s sport.

What sets these coaches apart, making them the best in the world and leaders in their fields? There is a notable lack of research on successful coaching experiences in China, highlighting a need for enrichment in coaching theory. This study aims to contribute to the global academic community by examining the experiences of gymnastics Olympic champion coaches within the Chinese context. Additionally, this study employs interview research, based on content spanning 2 years, allowing for a deeper exploration of cultural characteristics.

### 1.1 The perspective of coaching style

There are two primary coaching styles: democratic and autocratic. The democratic approach focuses on empowering athletes to set their own goals, training objectives, and game strategies, whereas the autocratic style underscores authority and unilateral decision-making. Additionally, the concept of servant leadership has gained attention in recent years. Research has extensively explored the impact of these coaching styles on athlete outcomes.

In individual sports like table tennis, democratic coaching behaviors have been linked to better coping mechanisms and emotional outcomes (González-García and Martinent, 2020). Rune et al. (2008) observed that athletes in adverse situations show a preference for democratic behaviors and social support more than in successful situations, suggesting that challenging scenarios might enhance players’ preference for a coaching style marked by significant task and relationship-oriented behaviors. Specifically, during periods of failure, players lean toward desiring more instructional and training-focused coaching, which directly contributes to task-oriented skill development. This approach has been recognized by elite athletes as crucial for improving performance and increasing the chances of success. Moreover, Rieke et al. (2008) introduced the concept of servant leadership in sports as a new paradigm for effective coaching, providing Christian sports coaches with a practical framework for their duties and responsibilities toward athletes and teams in a competitive environment (Vinson and Parker, 2021). According to Rieke et al. (2008), coaches who embrace servant leadership tend to foster athletes with healthier psychological profiles who also exhibit strong performance. However, in team sports, González-García et al. (2022) discovered that democratic coaching negatively impacts team task integration, while authoritarian coaching slightly improves social integration during competitions. Furthermore, mature and male athletes often prefer well-organized coaches with decisive decision-making skills, traits typically associated with autocratic coaching, as these athletes usually approach their sport with a higher degree of seriousness and view it as a structured organization (Breeman et al., 2014). Nevertheless, Woods et al. (2022) caution that an authoritative coaching style can lead to athlete burnout and psychological strain. Coaches adapt their leadership styles in response to specific circumstances. Throughout an athletic season, the dynamics of a coach’s role and interactions with athletes evolve, influencing factors such as individual win-loss records, team performance in dual and tournament competitions, practice intensity, the caliber of recent opponents, outcomes against formidable or weaker opponents, coaching tenure, and overall satisfaction with teammates, sport, and coaching. These elements collectively shape the leadership approaches employed by coaches (Turman, 2001).

### 1.2 The coach-athlete relationship

Both the coach’s and athlete’s behavior will influence each other’s perceptions and motivation levels (Jackson and Beauchamp, 2010; West, 2016). So, research involving coaches and athletes across various disciplines, competitive levels, and countries highlights the pivotal role of coach-athlete relationship quality in leadership and performance outcomes. Seiler (2006) identified this relationship as a critical determinant of competitive success. Kuhlin et al. (2019) analyzed 14 years of collaboration in figure skating, underscoring the influence of coach-athlete interactions on personal growth and career development. Drawing from foundational studies by Iso-Abola (1995) and Kelley et al. (1983), Jowett and colleagues emphasized the significance of these relationships in athletic performance and developed a comprehensive theoretical framework. This included the introduction of the 3C model (complementarity, co-orientation, closeness; Jowett and Meek, 2000, a coach-athlete relationship scale, and an integrated model linking relationship quality to individual and team performance (Jowett, 2007). Further, Jowett and Palmer (2010) demonstrated through surveys that negative aspects of the coach-athlete relationship, such as conditional or absent respect, can significantly impede athletic development and success by eroding trust, motivation, and performance (Mchenry et al., 2020). Additionally, research by Davis et al. (2021) and Ye et al. (2016) explored the mediating effects of interpersonal relationships on competitive outcomes within dyads. Minjung et al. (2018) advocated for a shift from a directive to a supportive coaching approach to foster these crucial relationships.

### 1.3 The aim of the current study

This study aims to explore a consistent coaching philosophy and approach of a distinguished Chinese coach through a case study, addressing the following reasons: Firstly, existing literature and theoretical frameworks exhibit limitations in thoroughly explaining the coaching philosophies of Chinese coaches, particularly within the context of China. Previous research has explored the effects of various coaching styles, such as democratic, servant, and authoritative, on athlete success. However, Chinese coaches often face the challenge of melding the seemingly contradictory styles of authority and democracy, a necessity arising from the unique structure of Chinese sports, where coaches are state-funded professionals rather than being privately hired by athletes. This setup mandates coaches to devise training strategies and objectives tailored to team needs, offering limited flexibility for athlete input, especially in areas aiming to improve professional competence and competitive experience. Additionally, the increasing focus on athletes’ physical and mental health in China necessitates a shift toward a more democratic coaching approach. Contrarily, elite athletes with international accolades may enjoy enhanced negotiation leverage, diverging from the predominant servant-style coaching observed in Western contexts. Furthermore, the authoritative 3C model of the coach-athlete relationship (Jowett and Meek, 2000), deeply ingrained in Chinese Confucian values, does not advocate for athlete subservience, suggesting that this model may not fully capture the essence of Chinese coaching philosophies. Previous research has predominantly utilized surveys to investigate the relationships among various factors, yet it has not adequately captured the nuanced experiences of coaches in cultivating world champions. These experiences are distinct and highly individualized. The efficacy of sustaining successful coaching practices over prolonged training periods varies, with coaching approaches often reflecting unique personal attributes. Consequently, interviewing elite coaches is essential to uncovering effective coaching philosophies and methods. While empirical insights derived from structured experiments are invaluable, qualitative insights can significantly complement these findings and deepen our understanding of sports performance (Greenwood et al., 2012). Furthermore, coaches play multifaceted roles, including planning, observing, and providing feedback (Kidman and Hanrahan, 2011). It is noteworthy that most prior studies have focused on specific coaching traits linked to athletic success through singular methodologies or theoretical frameworks, neglecting a comprehensive evaluation of the coaching strategies or qualities of champions. Given the limitations of existing measurement tools in capturing the full spectrum of coaching attributes, conducting interviews with coaches proficient in athlete support can reveal new and critical aspects of coaching effectiveness (Gould et al., 2002).

## 2 Methods

### 2.1 Participants

Qualitative research, as Creswell (2014) advocates, prioritizes the careful selection of participants to achieve a comprehensive understanding of the research problem and associated inquiries. The likelihood of success tends to increase with a coach’s experience, successful coaches often maintain their positions longer (Filho and Rettig, 2018). Thus, focusing on experienced and successful coaches is essential for this study. We chose to examine the coaching journey of Wang Guoqing, a distinguished coach at the Gymnastics Center of the General Administration of Sport of China, who boasts 20 years of coaching experience and has secured 8 gold medals in significant international competitions. Wang Guoqing, who holds a Doctorate in Sports Education from Beijing Sport University and is a second-level professor, brings a wealth of insight from his extensive career and demonstrates exceptional communication skills, essential for elucidating complex ideas and capturing critical information for this research.

Given the esteemed standing of our participant in Chinese Competitive Gymnastics and the in-depth nature of this case study, he consented to forego anonymity. This choice aligns with Wang’s philosophy of sharing knowledge, as evidenced in an early exchange of communication: “*I think sharing is the key to progress. Coaches are a philosophy or art of interacting with athletes. If we do not express our opinions, we will not be able to gain support or even doubt, and we will stop moving forward. The Olympic spirit has recently brought up the concept of greater unity, which I believe is also a strengthening of human understanding of competitive sports and the emphasis on communication between people. Coaching also requires a greater emphasis on exploring the best coaching methods with an open attitude toward the outside world* (Wang Guoqing, personal communication, 1 January 2022).”

### 2.2 Interview guide

Through the synergistic collaboration of a seasoned PhD in psychology from Beijing Sport University and an assistant professor with a specialization in sports psychology from Xiamen University’s Department of Physical Education, we have meticulously developed a semi-structured interview guide. This instrument is designed to explore the rich tapestry of world-class coaching success, with a keen focus on the competencies and collaborative dynamics between these coaches and their athletes. The guide is composed of a suite of open-ended questions crafted to elicit comprehensive responses that unveil profound insights. It has been subjected to a stringent review, meticulously examined by two distinguished coaches from the Chinese national team to ensure its pertinence and potency.

Following the execution of preliminary pilot interviews, which served as a trial run to assess the guidelines’ practicality and lucidity, we implemented a series of minor yet impactful refinements to the phrasing of the questions within the guide. These enhancements were made with the intent to sharpen the instrument’s precision and to catalyze a more profound and granular investigation into the lived experiences and viewpoints of our interviewees.

### 2.3 Data collection techniques

The data collection lasted for 2 years and 2 months. In order to comprehensively understand the interaction and coaching experience between Coach Wang (co-author of this study) and athletes, we continuously collected information through a combination of online and offline methods throughout the entire process. Initially, in order to gain a rough understanding of the interaction between Wang and the athletes, as well as the characteristics of his coaching, we developed an interview outline and the questions to be asked. Written informed consent from the coach has been obtained during the interview. In addition, these work hours span a long time because Coach Wang happens to have high-intensity training tasks, and he is unable to allocate a fixed amount of time, each lasting at least an hour or more, to share his coaching experience with the main author on the national gymnastics team. The information we obtain each time is very limited. It was not until February to March 2024 that Wang had more time to share his successful coaching experience with us. Therefore, we conducted targeted semi-structured interviews with Coach Wang. The main interview content includes: (1) What ability do you think you have to cultivate so many world-class champions? (2) What kind of interpersonal relationship do you think you have established with Olympic champions?

### 2.4 Data processing and analysis

Data analysis is conducted using the manifest qualitative content analysis approach, as outlined by Robson (2011, p. 469). The transcribed data is carefully reviewed multiple times to ensure a thorough understanding of its entirety. Using thematic coding techniques, we categorize and label the data to reflect relevant concepts. Codes that are similar or related then combines into distinct themes. The following sections outline the primary themes extract from the qualitative dataset, as originally presented by Currie and Oates-Wilding (2012). In line with the study’s objectives, theme, subtheme, and codes were inductively abstracted and condensed, focusing on the manifest content. Rigorous measures were taken to ensure that the categories exhibited internal homogeneity and external heterogeneity. The analysis was carefully executed by three of the authors, initially performed independently, followed by a collaborative discussion. The analytical process was iterative, characterized by a continuous oscillation between the data as a whole and its individual components. Notably, the coaches did not provide any feedback on the outcomes of the data analysis. Table 1 presents a representative example of the analytical procedure employed.

**Table 1:** The data analysis process.

Theme	Subtheme	Code
Training management and planning	International perspective and collaborative ability	Fully grasp the changes in international competition rules and ensure that the preparation work of athletes and coaching teams is consistent with international standards.
		It is difficult to make progress without going abroad to exchange and accept more advanced training concepts and methods
		Misunderstanding of rules leads to the failure of tactical arrangements in the competition
		Encourage and support athletes to participate in international competitions and tournaments to gain practical experience and improve their technical skills
		Pay attention to the latest developments in sports technology internationally, such as sports biomechanics, data analysis, psychological training, etc., and apply these technologies to the training of local athletes.
	Ability to control and regulate training loads	Gymnastics competitions have certain risks, and fatigue training poses safety risks
		Believe in the subjective feelings of athletes, and when they mention that they are tired, believe it.
		Always monitor the fatigue status of athletes.
Motivation and goal setting	Identifying athletes’ needs and transforming them into motivation	Engage in in-depth conversations with athletes to understand their interests, goals, and concerns
		Observe the behavior of athletes during training and competition to identify their needs and reactions. Understand the unique needs and motivations of each athlete.
		Customize training plans and support strategies to meet their personal goals.
		As a coach, embody the essence of passion and commitment, exemplifying the process of transforming enthusiasm and aspirations into tangible act
	Goal setting aligned with athletes’ abilities	Understand the skill level, physical condition, psychological state, as well as their strengths and weaknesses of athletes
		Ensure that the set goals are specific, measurable, achievable, relevant, and time bound
Interpersonal communication	Adopting authoritative democratic coaching style	Although we embody a father like strictness, it is crucial to remain approachable.
		To convey authority without succumbing to anger or arrogance, demonstrate your expertise and leadership with calmness and respect, ensuring that your authority is recognized without the need for outbursts or overt displays of power.
		Athlete’s compliance with coaches
		The democratic coaching style and friendly dialog allow athletes to openly express their thoughts and feelings.
	Establish hierarchical-style friendship	Obey the Chinese father hierarchical system
		Open Communication
		Mutual Trust
		Flexible Encouragement

### 2.5 Quality and rigor

Based on a previous article published in PSE (Qin et al., 2023), our method selection enhances the research quality within the framework of critical realism. We choose to demonstrate rigor through two main forms: rigorous reflection and rigorous methods. Strict reflection makes the transmission of research transparent, including the knowledge and theoretical foundation of researchers (Danermark et al., 2019). Through reflection, we acknowledge that our explanation is only a perspective, influenced by the participation of the national sports system and cooperation with elite athletes. A rigorous approach is achieved through genuine analysis and critical reflection from friends. In addition, we invited one national team athlete, one national team coach, and one psychology PhD to explain the main viewpoints and the reasons for their emergence in this study. The research results and process have been unanimously agreed upon.

## 3 Results

The inductive content analysis leads to that 6 subthemes (“international perspective and collaborative ability,” “ability to control and regulate training loads,” “identifying athletes’ needs and transforming them into motivation,” “goal setting aligned with athletes’ abilities,” “adopting authoritative democratic coaching style,” and “establishing hierarchical-style friendship”), and 3 themes (“training management and planning,” “motivation and goal setting,” “interpersonal communication”) are manifested (Table 1). An overarching theme “the successful experience of gymnastics world champion coach,” is derived from the analysis.

### 3.1 Training management and planning

The successful experience of world champion coaches in gymnastics underscores the critical importance of training management and planning, which can be divided into two sub-themes: international perspective and collaborative ability, and the ability to control and regulate training load.

#### 3.1.1 International perspective and collaborative ability

Our findings suggest that exemplary coaches should possess international perspective and collaborative ability, to be more specific, an international outlook, adopting advanced training methodologies and technologies through global exchanges and collaborations to bolster the team’s international competitiveness. This aligns with the principles of the Olympic Charter and the International Council for Coaching Excellence (ICCE), both dedicated to fostering the growth and global interchange among coaches. These entities underscore the significance of an international perspective and a collaborative ethos among world-class coaches. Lyle (2002) and Gould and Maynard (2009) posit that to thrive in the ever-evolving international sports landscape, outstanding coaches must continually update their knowledge and skills. Thoroughly understand the evolving rules of international competition and ensure that the athletes and coaching teams’ preparation aligns with global standards. Wang mentioned, “*I sometimes communicate changes in competition rules with Arturs Mituls, the chairman of the Men’s Artistic Gymnastics Committee of the International Gymnastics Federation*” “*I also often learn advanced training techniques and concepts, and adjust my own training plan at once*.” Wang also stressed, “*Gymnastics is developing, the difficulty is increasing, and the international rules are changing. If we do not keep up with the times, constantly learn, and improve ourselves, we will inevitably be eliminated by the tide of the times!”* For it is difficult to make progress without going abroad to exchange and accept more advanced training concepts and methods (Reade et al., 2008). If training methods and philosophies are not updated according to international trends, there is a risk of misunderstanding the rules, which could lead to tactical failures in competitions (Gould et al., 2002).

#### 3.1.2 Ability to control and regulate training loads

Our research indicates that the ability to control and regulate training loads is crucial for world-class coaches. Athletes participating in elite sports face high training loads and increasingly saturated competition schedules. The psychological and physiological fatigue caused by overtraining can lead to long-term poor performance (Smith, 2003). According to the International Olympic Committee, training load broadly includes rapid changes in training and competition loads, congested competition schedules, psychological load, and travel. Poor management of these factors is a major risk factor for injury (Soligard et al., 2016). This is especially true in gymnastics competitions, where inherent risks and fatigue during training pose significant safety concerns.

Our research also emphasizes that coaches should trust athletes’ subjective feelings toward fatigue, even though existing studies have proposed objective methods for quantifying exercise load, for example, the “training impulse” (TRIMP) which is consists of exercise intensity and duration calculated using the heart rate reserve method (Banister, 1991). However, ideally, athletes and coaches should match their perception of training load to achieve optimal adaptation (Pind and Mäestu, 2018). So, we hold Believe in the subjective feelings of athletes, and when they mention that they are tired, believe it, and always monitor the fatigue status of athletes.

### 3.2 Motivation and goal setting

The successful experience of world champion coaches in gymnastics also underscores the critical importance of motivation and goal setting, which can be divided into two sub-themes: identifying athletes’ needs and transforming them into motivation, and setting goals aligned with athletes’ abilities.

#### 3.2.1 Identifying athletes’ needs and transforming them into motivation

Our research reveals that adept coaches excel in discerning and linking athletes’ needs with their achievements, a key factor in engaging athletes and driving them toward their peak psychological and physiological states (Weinberg and Gould, 2015). This ability is rooted in the deliberate efforts of individuals to meet their needs (Cheng et al., 2023). Fulfilling athletes’ needs, particularly when it enhances their competitive performance or leads to outstanding results, serves as a significant motivational force in both training and competition (Vallerand, 2007). It should be noted, however, that the aspiration for achievement often operates on a subconscious level (McClelland et al., 1953), which requires coaches to explore and inspire.

Therefore, we believe it is necessary to focus on two key aspects. First, establish high-quality coach-athlete relationships and engage in in-depth conversations with athletes to understand their interests, goals, and concerns (Jowett and Cockerill, 2003). Second, a study of interviews with elite coaches found that, before intervening in performance issues, coaches must establish and maintain trust with athletes and their support networks. This ensures athletes feel safe discussing performance concerns and receive consistent and supportive information (Williams et al., 2023).

Wang Guoqing explained, “Only by establishing a realistic and tangible vision for each athlete can we maximize their inner potential and motivation for training. In the early stages of athletic training, their vision might be at a lower level, such as obtaining material rewards. As they progress and win more competitions, their vision gradually shifts toward higher levels, such as winning respect, proving oneself, and self-actualization. Of course, the pursuit of fame, fortune, and material possessions still exists. In reality, various human needs coexist, but at different times, different needs take precedence, and people prioritize meeting their most pressing needs.”

The concept outlined above is corroborated by Goldthorpe (1987), whose research into social mobility and class structure in the UK underscores the impact of economic foundations on various social strata, positing that economic standing significantly influences one’s social status and interests.

On the other hand, as a coach, it is important to be a model of passion and commitment, demonstrating how to translate passion and goals into action. This approach is supported by many researchers (Martens, 2012; Whitmore, 2017).

#### 3.2.2 Goal setting aligned with athletes’ abilities

We find that skilled coaches can set goals based on the abilities of athletes, which can enable them to impart knowledge on skill development and prepare athletes to achieve optimal performance (Gould et al., 2002; Johnson et al., 2011; Kuma, 2019). It’s also supported by Gearity and Murray (2011), they identify five themes from athletes’ self-reports: inadequate coach instruction, indifference, unfairness, inhibition of athletes’ psychological skills, and athlete coping. Two of these themes, inhibition of athletes’ psychological skills and coping, are closely related to psychological structure and are discussed in this article. The theme of inhibiting athletes’ psychological skills includes descriptions of poor coaching practices that can distract attention, generate self-doubt, weaken motivation, and divide the team. The theme of athlete coping describes how athletes manage and respond to poor coaching.

In addition, the present study finds that a great Olympic coach should ensure that the set goals are specific, measurable, achievable, relevant, and time bound. As scholars emphasize the importance of setting SMART goals to provide direction and focus for athletes (North et al., 2021).

### 3.3 Interpersonal communication

Interpersonal communication is vital for world-class coach including the ability adopting authoritative democratic coaching style and establishing hierarchical-style friendship.

#### 3.3.1 Adopting authoritative democratic coaching style

Our research has found that using an authoritative democratic coaching style is an important coaching experience. The authoritative democratic coaching style is a hybrid approach to leadership that combines the assertiveness and clear direction of authoritative coaching with the inclusiveness and participatory nature of democratic coaching. In this style, the coach maintains a strong sense of authority and control, which serves as the foundation for fostering a collaborative and engaging environment.

In this coaching style, the coach sets clear goals and expectations for their team members, but also encourages open communication and feedback. As Jones et al. (2019) note, this approach allows for a more collaborative environment where team members feel heard and valued. The coach listens to the opinions and suggestions of their team members and considers them when making decisions. They also provide guidance and support to help their team members develop their skills and achieve their goals.

This coaching style fosters a positive and collaborative team environment, where team members feel valued and respected. It promotes trust and accountability, as team members are encouraged to take responsibility for their actions and work together toward a common goal. Overall, the authoritative democratic coaching style is effective in creating a high-performing team that is motivated, engaged, and committed to achieving success (Jones et al., 2019).

In response to the question, “what kind of interpersonal relationship do you think you have established with Olympic champions?” Coach Wang in our study once replies: maintain a disciplined and approachable demeanor, similar to a father’s image. To achieve self-reliance without anger. Subsequently, the researchers questioned whether this authority or authority would create a fear that would be detrimental to the success or mental health of athletes? Wang clarified, “Although we embody a father like strictness, it is crucial to remain approachable. The democratic coaching style and friendly dialog allow athletes to openly express their thoughts and feelings.”

#### 3.3.2 Establish hierarchical-style friendship which is a positive coach-athlete relationships

Our research indicates that elite coaches prioritize fostering positive coach-athlete relationships characterized by Obeying the Chinese father hierarchical system, open communication, mutual trust, and encouragement. These relationships are foundational to improved athletic performance (Weinberg and Gould, 2015). Jowett and Cockerill (2003) find that Olympic medalists consistently identified open communication, mutual trust, and encouragement as critical components in building positive coach-athlete relationships.

##### 3.3.2.1 Obey the Chinese father hierarchical system

We find that great coaches need to maintain confidence and authority, otherwise it is difficult for athletes to trust them. The hierarchical structure within the Chinese national sport system significantly influences the behavior and compliance of athletes and coaches. This system, often referred to as the “Whole-Nation system,” is characterized by its centralized structure, medal-oriented focus, and semi-closed environment (Ge et al., 2016). The legitimacy of coaches demanding obedience from athletes can be understood through several key points:

Centralized Structure and Medal Orientation: The centralized structure ensures athletes are trained under a unified methodology, crucial for high performance and winning medals. This system places immense pressure on athletes, with coaches playing a pivotal role in guiding them. The hierarchical nature necessitates a clear chain of command, where athletes follow coaches’ directives to achieve collective goals (Ge et al., 2016).
Psychological Training and Sociocultural Meridians: Psychological training is deeply rooted in sociocultural meridians, including cultural inheritance and traditional beliefs such as “harmony with differences” and the balance between Confucianism and Taoism (Si et al., 2011). These elements emphasize respect for authority and hierarchical relationships, integral to the coach-athlete dynamic in China. Integrating these characteristics into training fosters discipline and respect, legitimizing coaches’ authority.
Cultural and Psychological Integration: Integrating cultural and psychological factors into training is crucial for athletes’ overall development. The hierarchical system, aligning with traditional Chinese values, provides a stable framework for growth. Coaches, by embodying these values and maintaining structure, help athletes navigate elite sports pressures and achieve their potential (Si et al., 2011).

In conclusion, the hierarchical system within the Chinese national sport system is both a cultural norm and a practical necessity for achieving excellence in sports. Coaches’ demands for obedience are legitimized by the need for a centralized, disciplined, and culturally coherent training environment. This system ensures athletes are well-prepared to meet international competition challenges and achieve success on the global stage.

##### 3.3.2.2 Open communication

Open communication is essential for preventing misunderstandings and conflicts due to insufficient communication. It facilitates the timely and effective resolution of issues arising during training and competition, thereby enhancing professional performance and promoting the personal growth of athletes. Moran and Megan (2010) even propose an innovative coaching approach that emphasizes the autonomy and professional development of coaches through four core practices: listening, expressing, designing, and supporting. They advocate for a faultless environment and strengths-based improvement, demonstrating the transformative power of dialog in educational improvement.

In China’s competitive sports culture, even Olympic champions must respect the authority of their coaches, who hold significant dominance during training. However, elite athletes often have a clear understanding of their needs. As Olympic champion Xu Mengtao articulated, athletes can fully follow the coach’s guidance from 0% to 99%, but to achieve the final 1% to reach 100%, athletes must understand their own strengths and weaknesses. They should communicate effectively with their coaches about their training programs, even suggesting amendments based on their insights. This perspective is supported by a comprehensive meta-analysis (Theeboom et al., 2014).

However, the traditional Chinese education system, rooted in Confucianism, emphasizes “respect for teachers and valuing their teachings.” Teachers are seen not only as knowledge transmitters but also as moral guides. Students are expected to treat their teachers with utmost respect and diligently learn from them. In this cultural context, coaches are akin to teachers, enjoying absolute respect and generally holding the view that their authority should not be questioned. Coaches who practice open communication are rare. Most prefer to maintain their positions and perspectives, even when faced with challenges, and expect athletes to reflect on their own shortcomings rather than questioning the coach’s methods.

Coach Wang’s coaching philosophy commands respect and recognition, not only because he genuinely respects his players but also because he embraces their questioning. Wang states, “I will correct what my players say I really did wrong.” His excellent communication skills enable him to address the team’s issues promptly. Wang emphasizes the importance of communication, stating, “I think it’s very important to communicate with the athletes in my group. In addition to teaching them the essentials of movement in training, we often communicate with each player individually, allowing them to speak freely and respecting their opinions. For incorrect ideas and demands, I educate them through persuasion and guidance, and the effect is very good!” This approach has resulted in increased respect and trust from the players. By fostering an open dialog with his players, Wang has cultivated a strong sense of team cohesion and trust, solidifying the Chinese gymnastics team’s status as the ace of China’s sports corps.

##### 3.3.2.3 Mutual trust

Mutual trust is the cornerstone of the coach-athlete relationship. Its supported by Schiemann et al. (2019) and Tiitu (2017). Trust enables coaches and athletes to collaboratively develop and adapt training programs to achieve optimal performance. For instance, the collaboration between renowned track and field coach Alberto Salazar and Olympic champion Mo Farah exemplifies a high level of mutual trust. During Farah’s training, Salazar continuously adjusted the training intensity and methods based on Farah’s feedback, ultimately contributing to Farah’s outstanding achievements.

Unlike the traditional emphasis on high intensity and large training volumes to produce results, Coach Wang advocates for flexible training methods based on trust. He stated, “I always trust my athletes, and when they tell me they are not in good health and wish to reduce, modify, or even rest, I usually agree. Sometimes they voluntarily increase their training despite fatigue; in such cases, I insist that they rest to prevent injuries from overtraining. It has been proven that each time I support their adjustments and rest, they work harder and often exceed their previous training achievements.”

With Coach Wang’s trust, the athletes feel comfortable expressing themselves honestly, rather than lying to secure more vacation time. They are not only transparent about their rest needs but also proactively seek Coach Wang’s advice on crucial decisions during training and competitions. This relationship of trust allows the players to fully embrace Coach Wang’s guidance, enabling them to commit to their training without resistance.

This coach-athlete relationship, founded on mutual trust, not only enhanced the athletes’ training outcomes but also strengthened team cohesion. Ultimately, this contributed to the improvement of the team’s overall performance.

##### 3.3.2.4 Flexible encouragement

Coach encouragement, as the primary form of social support, plays a critical role in promoting athlete success. The motivational model of the coach-athlete relationship emphasizes the importance of a coach’s encouragement and support in enhancing athletes’ motivation and performance. Encouragement not only boosts an athlete’s confidence but also stimulates their potential (Gilbert and Trudel, 2004). Coaches must recognize even small improvements in their players (Gould et al., 2002). Former USA Gymnastics coach Bella Karolyi is known for his motivational training methods, which inspired athletes to strive for success in international events through constant encouragement and praise.

Coach Wang stated, “In my coaching process, consistent encouragement and praise are given throughout the training. Acknowledgement and affirmation are especially provided when athletes exhibit an indomitable spirit, courage, and resilience in the face of setbacks.” Encouragement from the coach helps athletes to face challenges bravely and make progress. However, when an athlete achieves a major accomplishment and begins to show slackness, the coach should withhold encouragement and instead help the athlete adjust their mindset by motivating them to pursue higher goals. As Coach Wang said, “I usually give them the most praise and encouragement when they are facing challenges, and remind them to refrain from arrogance after they have succeeded.”

This dynamically adapted encouragement strategy not only motivates athletes at critical moments but also keeps them grounded and humble as they achieve success and continue to pursue excellence. By establishing a coach-athlete relationship based on open communication, mutual trust, and continuous encouragement, it is possible to effectively enhance an athlete’s overall performance and mental fitness.

## 4 Discussion

The study identifies three key themes in the successful experiences of gymnastics world champion coaches: “training management and planning,” “motivation and goal setting,” and “interpersonal communication.” These themes are reflected in the following abilities: (1) “international perspective and collaborative ability,” (2) “ability to control and regulate training loads,” (3) “identifying athletes’ needs and transforming them into motivation,” and (4) “goal setting aligned with athletes’ abilities.” These aspects are validated by current research and contribute a new perspective to the existing body of knowledge. Additionally, the study introduces two innovative concepts: (5) “adopting an authoritative democratic coaching style” and (6) “establishing hierarchical-style friendships.” These innovations provide a fresh perspective on the successful experiences of coaches, offering valuable insights into effective coaching practices to the world.

To be more specific,

1. “International perspective and collaborative ability”

Currie and Oates-Wilding (2012) believe that knowledge of Sport and Focus on Needs of Athlete is one of the most vital factors Olympic coaches attribute to their success. So, coach Wang points out that actively participating in international competitions and learning about advanced technologies and their applications are essential experiences for a successful coach. For (1) participating in international competitions and tournaments will gain athletes’ practical experience and improve their technical skills (Gould and Carson, 2008). (2) Stay attuned to the latest developments in international sports technology, including sports biomechanics, data analysis, and psychological training, especially in the era of digital sports transformation driven by AI, VR, AR, and DV (Gould and Maynard, 2009) and apply these technologies to the training of athletes, which is the top priority of future Olympic preparations (Cossich et al., 2023). Some scholars have proposed the trend of intelligent transformation of training methods based on the intelligent data analysis methods currently used in the field of intelligent sports training (Rajšp and Fister, 2020). For example, an increasing number of “sports vision training” practices rely on the idea that practicing high-demand visual perception, cognition, or eye movement tasks can enhance the ability to process and respond to visual stimuli, thereby improving sports performance (Appelbaum and Erickson, 2016).

1. “Ability to control and regulate training loads”

The ability to control and regulate training loads not only aids in preventing fatigue, thereby extending the competitive careers of athletes, but also enhances the efficiency of training sessions. Effective regulation of training loads necessitates that coaches possess a comprehensive understanding of athletes’ perceptions of the imposed workload, the importance of sufficient rest, and the physiological changes stemming from consecutive training sessions (Impellizzeri et al., 2004; Buchheit et al., 2013).

As Wang mentioned, *“the fatigue of athletes in gymnastics is twofold—physical fatigue and psychological fatigue, which is different from other sports. In fact, most injuries of gymnasts are caused by fatigue, and in situations of abundant physical strength and good physical and mental state, they are less likely to be injured” “Physical fatigue is easy to recover from, while psychological fatigue is more challenging. In severe cases, even top athletes may not dare to perform difficult movements, and improper control of training volume can also lead to this situation.”*

“*Now our group has 6 team members, and there are 6 training plans with different training contents, which may be different from group sports. Usually, their training starts at the same time, but the end times often* var*y. One of my athlete’s training characteristics are high intensity, short duration, and few but precise. And another one entered the training state slowly, so his intensity was arranged to be moderate, relatively long, and lukewarm. he often ended the training class last.*”

1. “Identifying athletes’ needs and transforming them into motivation”

High intensity training is full of pressure and frustration, which requires strong mental motivation to complete. Need to be able to motivate and highly connect with personal needs. As Mageau and Vallerand (2003) proposed a motivational model for the coach-athlete relationship, emphasizing the role of communication and understanding in establishing effective relationships. Coaches must observe athletes’ behavior during training and competition to identify their needs and reactions. Understanding the unique needs and motivations of each athlete is crucial. Wang Guoqing stated, “Honors, rewards, and benefits are the most effective motivators. I often ask athletes why they practice gymnastics, what their dreams are, or what they need the most. Then, I help athletes establish a vision map, showing what they can achieve once they succeed or win a championship.” Furthermore, according to the self-determination theory, individuals are more motivated to pursue their goals when their basic needs are met, leading to greater focus and engagement (Deci and Ryan, 2000). This means that the satisfaction of needs and success are mutually reinforcing. According to Maslow’s hierarchy of needs theory, the satisfaction of different levels of needs corresponds with success at different levels.

1. “Goal setting aligned with athletes’ abilities”

It’s also supported by the scholars, the reason may be that, on the one hand, humans are purposeful agents who act to meet their needs. However, the human soul is a mosaic composed of multiple selves and conflicting needs. Scholars have proposed a psychological mechanism for resolving this conflict and contradiction, namely “goal persistence.” “Goal persistence” refers to persisting in pursuing the original intention despite unexpected changes that may make the intention less ideal (Cheng et al., 2023). In addition, Locke and Latham (2002) summarize 35 years of empirical research on goal setting theory and found that emphasizing the importance of goal setting for individual effort and focus. On the other hand, Set goals based on athletes’ competence is essential, for both self-efficacy theory and self-determination theory believe that when the difficulty of a task exceeds the level that an individual can achieve through effort, it is easy for the individual to lose confidence and motivation (Bandura et al., 1997; Deci and Ryan, 2000).

As stated by Wang, “*the setting of goals is very important, and I have divided them into three stages: short-term, medium-term, and long-term. However, tailored plans and goals tailored to each athlete’s characteristics are the most crucial. The goals I set during training are not too high, and through hard work, they can achieve them. When they achieve the established goals, they have more confidence to challenge new goals. If the goal is set too high, it will be difficult for athletes to achieve it through hard work, which indicates that the plan formulated by the coach is wrong and unrealistic, and more importantly, it will undermine the confidence of the athletes.*”

Mastering these skills presents a formidable challenge, necessitating coaches to exhibit two essential qualities:

1. “Adopting authoritative democratic coaching style”
2. “Establishing hierarchical-style friendship”

For the first, the establishment of a hierarchical-style rapport is vital, enabling coaches to assert authority within the context of China’s national structure and traditional values, akin to a paternal figure, contrasting with Western egalitarian norms. Second, implementing an authoritative democratic coaching approach is crucial, where coaches lead with confidence and authority while allowing athletes the liberty to voice their thoughts and feedback. This strategy aims to spur innovation, instill a sense of responsibility in team members, and foster collaborative achievement of shared objectives. As repeatedly highlighted by Wang, a champion coach’s demeanor, while non-confrontational, should exude a natural, commanding presence that commands respect. This dual approach—hierarchical-style rapport and authoritative democratic coaching—enriches the dialog on coaching methodologies and the dynamics of coach-athlete interactions, offering fresh perspectives and strategies for international coaching research. The introduction of two concepts—authoritative democratic coaching and hierarchical-style friendship—is driven by several considerations. Firstly, champion coaches must possess decision-making authority, exhibiting strength and decisiveness, as highlighted by Wang, who emphasized the critical importance and influence of gymnastics in the Olympics. He argued that to secure a team championship, coaches must demonstrate boldness and authoritative leadership (Rune et al., 2008). Wang also noted the psychological dependence of gymnasts on their coaches, underscoring the necessity for a coach who commands respect and trust. He further posited that athletic performance is key to gaining team members’ respect, and that authoritative leadership requires a range of supporting skills. Additionally, the study suggests that the adoption of an authoritative leadership style is shaped by China’s national sports system and Confucian culture. Specifically, the structure of China’s competitive sports, being a state system, differs fundamentally from the market-driven model prevalent in the West. In China, coaches and athletes serve the nation’s interests, with the government covering their salaries and coaches enjoying a status akin to government officials. This establishes a hierarchical dynamic with athletes, contrasting with the Western model where athletes may hire and dismiss coaches based on personal preference, leading to a more egalitarian coach-athlete relationship. Wang remarked that coaches, being employees of the General Administration of Sport of China, cannot adopt a servile approach. This distinction necessitates a balance between authority and rapport in coaching strategies.

Cultural distinctions significantly influence coaching approaches in Western countries and China. Western coaches tend to favor a more democratic style, emphasizing the importance of building relationships, valuing athletes’ participation and autonomy for their motivation and development (Jowett, 2007; Becker, 2009), and leveraging personal experiences to derive insights (Côté and Sedgwick, 2003). This perspective views the coach’s role as not merely instructive but as facilitators of athlete independence, decision-making involvement, and critical thinking skills (Côté and Gilbert, 2009). Conversely, Chinese coaching practices, deeply rooted in Confucian traditions, often embody a more paternalistic approach. Yao (2000) notes that Chinese coaches, guided by Confucian ethics, assume a fatherly role, expecting rigorous adherence from athletes, in line with the “Five Virtues” of societal expectations. Reflecting these cultural norms, Wang et al. (2014) adapt the coach-athlete relationship scale to include elements of athlete obedience. Wang further illustrates this dynamic by comparing his longstanding relationships with athletes to that with his own son, emphasizing the duration and depth of these connections. He argues that a top-tier gymnast typically remains with the same coach until retirement, underscoring the rarity of coach changes, except in cases of lesser skilled coaches. Frequent changes in coaching affiliations, Wang suggests, correlate with a higher likelihood of an athlete’s premature exit from the sport, highlighting the stability provided by enduring coach-athlete partnerships in contributing to sustained success in gymnastics.

To make informed decisions and successfully steer athletes toward competitive excellence, coaches must continually gather information about athletes’ physiological, psychological, and competitive conditions through engaging in democratic coaching practices and fostering open communication. Central to this approach is the establishment of positive, cooperative relationships characterized by transparent communication, mutual trust, and support. Specifically, a culture of openness, where individual self-expression leads to reciprocal sharing of personal experiences (Miller, 1990) lays the groundwork for building trust, enhancing understanding, and fostering a deep connection between athletes and coaches. This trust empowers athletes to fully embrace coaching guidance, thereby boosting their commitment and effort. Such relationships and effective communication not only strengthen team unity and collaboration but are also pivotal for the training success and overall performance of Olympic champions. Wang has highlighted *the significance of communication, noting that even highly skilled coaches may falter if they seldom engage with their team. A lack of open dialog can deter athletes from sharing vital insights about their physical and mental states, potentially leading to training approaches that ignore individual needs, thereby causing friction and, more critically, increasing the risk of injuries*. According to Knudson and Morrison (2002), a key responsibility for coaches is to clearly articulate necessary adjustments for performance enhancement. Clear communication enables athletes to understand coaching strategies and goals, amplifying their motivation and zeal for training. Moreover, effective communication among coaching staff fosters a collaborative environment, aligning objectives, providing precise feedback, and offering the support needed to navigate challenges and unlock potential (Fletcher and Arnold, 2011). Positive, collaborative relationships also play a crucial role in resolving conflicts, managing psychological challenges (Kavussanu et al., 2008), and addressing career-related concerns between coaches and athletes (Stambulova and Wylleman, 2019).

## 5 Strengths, limitations, practical application, and possible directions for future research

### 5.1 Strengths

Innovative Academic Perspectives: We introduced the concepts of “authoritative democratic coaching style” and “hierarchical-style friendship,” enriching the discourse on coaching styles and coach-athlete relationship theories. This contribution offers fresh perspectives and methodologies for the field of international coaching research. In addition, following Hodgson et al.’s (2017) recommendation to concentrate on a singular sport, this study’s exploration of gymnastic coaching offers specific environmental insights, minimizing the potential interference from other sports.

### 5.2 Limitations

Although the findings might be relevant to other Olympic disciplines, further sport-specific research is warranted. Reliance on Self-Reports: The study’s sole dependence on coaches’ self-reports introduces the risk of self-deception bias, as highlighted by Colbert et al. (2012). Future research should aim to corroborate and expand upon our results by incorporating observations from athletes, considering the relational and interpersonal nature of coaching.

### 5.3 Practical application

This study provides significant contributions to the field of sports coaching by identifying three key themes in the successful experiences of gymnastics world champion coaches: “training management and planning,” “motivation and goal setting,” and “interpersonal communication.” These themes are further elucidated through the following abilities: “international perspective and collaborative ability,” “ability to control and regulate training loads,” “identifying athletes’ needs and transforming them into motivation,” and “goal setting aligned with athletes’ abilities.” These aspects are validated by current research, thereby enriching the existing body of knowledge with a new perspective.

Moreover, the study introduces two innovative concepts: “adopting an authoritative democratic coaching style” and “establishing hierarchical-style friendships.” These novel insights provide a fresh perspective on the successful experiences of coaches, offering valuable guidance for effective coaching practices. By integrating these innovative approaches, the study not only enhances our understanding of elite coaching strategies but also offers practical applications for coaches aiming to achieve excellence in their respective sports.

In summary, this research bridges the gap between theoretical knowledge and practical application, providing a comprehensive framework for understanding and implementing successful coaching practices. The findings have the potential to influence coaching methodologies globally, fostering the development of resilient, motivated, and high-performing athletes.

### 5.4 Possible directions for future research: future research directions

(1) Building on the strengths and addressing the limitations of this study, future research should focus on several key areas to further advance the field of sports coaching. (2) Sport-Specific Research: While this study provides valuable insights into gymnastics coaching, there is a need for sport-specific research across other Olympic disciplines. (3) Longitudinal Studies: Conducting longitudinal studies will allow researchers to track the long-term impact of coaching strategies on athlete performance and development. This will help in identifying which coaching practices are most effective over time and how they contribute to sustained athletic success. (4) Cross-Cultural Comparisons: Given the global nature of sports, it is essential to explore how different cultural contexts influence coaching practices and athlete responses. Cross-cultural studies can provide insights into the adaptability and effectiveness of the “authoritative democratic coaching style” and “hierarchical-style friendship” in various cultural settings.

## 6 Conclusion

The inductive content analysis of this study has identified six subthemes and three themes that encapsulate the successful experiences of gymnastics world champion coaches. The subthemes include “international perspective and collaborative ability,” “ability to control and regulate training loads,” “identifying athletes’ needs and transforming them into motivation,” “goal setting aligned with athletes’ abilities,” “adopting authoritative democratic coaching style,” and “establishing hierarchical-style friendship.” These subthemes are organized under the main themes of “training management and planning,” “motivation and goal setting,” and “interpersonal communication,” all contributing to the overarching theme of “the successful experience of gymnastics world champion coach.”

### 6.1 Training management and planning

The study underscores the critical importance of training management and planning, highlighting the need for an international perspective and collaborative ability, as well as the ability to control and regulate training loads. Coaches must stay abreast of global competitive sports trends and adapt advanced training methodologies to enhance their team’s international competitiveness. Effective regulation of training loads is crucial to prevent fatigue and injuries, ensuring athletes’ long-term performance and career longevity.

### 6.2 Motivation and goal setting

Successful coaches excel in identifying athletes’ needs and transforming them into motivation. They establish high-quality coach-athlete relationships (Cook, 2019), engage in in-depth conversations to understand athletes’ interests and goals, and set specific, measurable, achievable, relevant, and time-bound (SMART) goals aligned with athletes’ abilities. This approach fosters a sense of purpose and direction, enhancing athletes’ focus and engagement.

### 6.3 Interpersonal communication

Interpersonal communication is vital for world-class coaches, encompassing the adoption of an authoritative democratic coaching style and the establishment of hierarchical-style friendships. The authoritative democratic coaching style combines assertiveness with inclusiveness, fostering a collaborative and engaging environment. Establishing hierarchical-style friendships, characterized by open communication, mutual trust, and encouragement, is essential for building positive coach-athlete relationships.

The study introduces two innovative concepts: “adopting an authoritative democratic coaching style” and “establishing hierarchical-style friendships.” These concepts provide fresh perspectives on effective coaching practices, emphasizing the importance of balancing authority with collaboration and fostering strong, trust-based relationships with athletes. By integrating these approaches, coaches can create a high-performing team environment that is motivated, engaged, and committed to achieving success.

In conclusion, this research bridges the gap between theoretical knowledge and practical application, offering valuable insights into the successful experiences of gymnastics world champion coaches. The findings have the potential to influence coaching methodologies globally, fostering the development of resilient, motivated, and high-performing athletes. Future research should focus on sport-specific studies, longitudinal analyses, and cross-cultural comparisons to further advance the field of sports coaching and validate the effectiveness of these innovative coaching strategies.

## Data availability statement

The raw data supporting the conclusions of this article will be made available by the authors, without undue reservation.

## Ethics statement

The studies involving humans were approved by the Ethics Committee of the Department of Physical Education, Xiamen University. The studies were conducted in accordance with the local legislation and institutional requirements. Written informed consent for participation in this study was provided by the participants’ legal guardians/next of kin. Written informed consent was obtained from the individual(s) for the publication of any potentially identifiable images or data included in this article.

## Author contributions

XL: Writing – review & editing, Writing – original draft, Visualization, Validation, Supervision, Software, Resources, Project administration, Methodology, Investigation, Funding acquisition, Formal analysis, Data curation, Conceptualization. XW: Validation, Writing – review & editing, Formal analysis. HQ: Formal analysis, Writing – review & editing. SM: Formal analysis, Methodology, Supervision, Writing – review & editing, Validation, Resources. GW: Writing – review & editing, Writing – original draft, Supervision, Methodology, Formal analysis, Conceptualization.
